# Preparation and Filling of a Simple Cavity in the Right Superior Cuspid, Posterior Proximal Surface

**Published:** 1860-05

**Authors:** J. H. Paine

**Affiliations:** Franklin, O.


### PREPARATION AND FILLING OF A SIMPLE CAVITY IN THE RIGHT SUPERIOR CUSPID, POSTERIOR PROXIMAL SURFACE.

BY DR. J. H. PAINE, FRANKLIN, O.

[Read at a meeting of the Mad River Valley Association.]

The great importance of preserving the human teeth is not questioned, we presume, by any one; and as the *cuspidati* occupy a more prominent position in the jaws than any others of the set, the preservation of them becomes matter of the utmost importance. We find them most frequently decayed on the posterior proximal surface, owing, no doubt, to the form of the tooth itself; the posterior proximal surface being more favorable for the retention of substances that cause or superinduce caries.

Our patient is requested to take the chair, and upon close examination with mouth glass and excavators of untempered fine points, we find, among others, that the right superior cuspid is decayed upon its posterior proximal surface. We will try to give our plan of filling such a cavity. The first thing to be done is to make space sufficient for the proper excavations, preparations, and plugging of same. The writer is aware of the timidity that frequently deters young operators from making that necessary space in order to the proper introduction and consolidation of a good filling. But let us no longer be afraid to do right. Let us have space, and a plenty of it, so that we have room to rotate the shanks of our excavators, pluggers and burnishers. The space should be made with cutting instruments. The cutting should be confined to the cuspid alone, unless the anterior proximal surface of first right superior bicuspid is decayed; in which case, a file, cut upon both its sides, may be used. But if the decay is confined alone to the cuspid, a safe-sided file should be used, the plain or safe side being carefully kept towards the bicuspid, not in the least wise scratching or cutting its healthy surface. The space should be greater at the palatial border of the cavity than at its labial. The file should occasionally be wet in tepid water, to prevent its becoming choked by the cuttings from the tooth.

When space enough is acquired, and the new surface well polished with files and Scotch stone, we proceed to the removal of caries from the cavity. This, we think, is best done with the ax and hoe-shaped excavators, having several of such at hand, with the blades of different lengths and breadths, its removal is soon effected.

We now proceed to the preparation of the cavity for the proper retention of the filling; for it is seldom that the removal of the decay is all that is necessary to be done in order to fill a cavity properly. First, then, retaining points must be made. These may be formed most easily with the ax-shaped excavators. There should be a dozen or more differently sized blades at hand, some long, with narrow cutting points, and some of different lengths, with different breadths of blades, and of course they should be sharp, and of good unyielding temper. A stroke is not always the best motion to cut properly within the cavity with the dentist's ax, though it may be with the woodman's. A semi-rotary movement, given with a keen blade, cuts quickly and more correctly the part desired to be taken away than any other movement that we know of at present. The shovel-shaped excavator is also needed in cutting from the hand, in the direction of the root of the tooth. This should be square on its edge, the blade set at an angle of about 45° with the shaft. The hoe-shaped excavator is needed, sometimes, in cutting towards the hand of the operator, or apex of the tooth. *The cavity must be retentive ;*—at least as large as its orifice, and we prefer it a little larger.

It now having the right form, we proceed to fill it with gold. We prefer " adhesive" gold foil, of any reliable brand. We use strips, preparing them by folding a sheet into about four thicknesses, and cutting them with foil scissors in widths of about two lines. The cavity is now to be wiped out thoroughly by bits of cotton, and a clean napkin placed immediately behind the cavity to be filled, and retained at its place by the forefinger and thumb of the left hand of the operator, to prevent the breath of the patient or saliva from the buccal gland insinuating itself into the cavity, preventing the successful condensation and solidification of the plug. We have our pluggers and strips at hand, and proceed at once to inserting the filling. The plugger must have sharp points. We attach the end of one of the strips to the points of our plugger, and convey it at once to a retaining point, or pit, and fasten it there by compression, and continue adding by links or folds, compressing thoroughly the part carried to the bottom of the cavity, leaving the outer end of link uncompressed for the present. We continue laying link upon link, until the cavity is full—*solidly full ;* then, beginning again at the margin of the filling, we condense with the same instrument, the outer ends of the links that stand upon the border, and approaching the center. If not enough of gold has been introduced to make it solid and full, we take an ax, of quite a long blade, and cut a deep gash in the center of the plug, and insert gold into it until it is full and solid. Then with a larger faced condenser, of four or more points, we go over the filling as before, beginning at the margin. When indentations can no longer be made by compression, we proceed to file away the excess of gold. This is best done by files of fine cut, wetting the file occasionally in tepid water and passing quickly and lightly over the whole surface of the filling, until it is perfectly level with the walls of the cavity.

Many have failed at this point, who did all the work of putting in a filling well, because they were remiss here. It will not do to leave the plug projecting beyond the walls of an approximal cavity; the act of mastication brings substances in contact with the filling, exerting a lateral pressure that, sooner or later, displaces the filling. The plug being now dressed even with the wall of the cavity, we proceed to polish it and the surface of the plugged tooth, with slips of Scotch stone, dipped occasionally in water. When perfectly smooth, burnishers are lightly and quickly passed over the whole surface, until in our judgment, it is perfectly smooth. The plug is now completed.

There may be a little tenderness for several days, when cold or hot substances come in contact with the new surface; but as soon as the new surface within the tooth is re-ossified, it will have passed away.

